# Acidic residues in the membrane-proximal stalk region of vaccinia virus protein B5 are required for glycosaminoglycan-mediated disruption of the extracellular enveloped virus outer membrane

**DOI:** 10.1099/vir.0.009092-0

**Published:** 2009-07

**Authors:** Kim L. Roberts, Adrien Breiman, Gemma C. Carter, Helen A. Ewles, Michael Hollinshead, Mansun Law, Geoffrey L. Smith

**Affiliations:** Department of Virology, Faculty of Medicine, Imperial College London, St Mary's Campus, Norfolk Place, London W2 1PG, UK

## Abstract

The extracellular enveloped virus (EEV) form of vaccinia virus (VACV) is surrounded by two lipid envelopes. This presents a topological problem for virus entry into cells, because a classical fusion event would only release a virion surrounded by a single envelope into the cell. Recently, we described a mechanism in which the EEV outer membrane is disrupted following interaction with glycosaminoglycans (GAGs) on the cell surface and thus allowing fusion of the inner membrane with the plasma membrane and penetration of a naked core into the cytosol. Here we show that both the B5 and A34 viral glycoproteins are required for this process. A34 is required to recruit B5 into the EEV membrane and B5 acts as a molecular switch to control EEV membrane rupture upon exposure to GAGs. Analysis of VACV strains expressing mutated B5 proteins demonstrated that the acidic stalk region between the transmembrane anchor sequence and the fourth short consensus repeat of B5 are critical for GAG-induced membrane rupture. Furthermore, the interaction between B5 and A34 can be disrupted by the addition of polyanions (GAGs) and polycations, but only the former induce membrane rupture. Based on these data we propose a revised model for EEV entry.

## INTRODUCTION

*Vaccinia virus* (VACV) is the prototypical member of the genus *Orthopoxvirus* of the *Poxviridae* (Moss, 2007). Like other poxviruses, VACV replicates in the cytoplasm, encodes many transcriptional enzymes (Broyles, 2003) and produces multiple forms of virion (Smith *et al.*, 2002; Condit *et al.*, 2006; Roberts & Smith, 2008). The first infectious virion produced is called intracellular mature virus (IMV). IMV represent the majority of infectious progeny and each virion is surrounded by a single lipid envelope (Dales & Siminovitch, 1961; Hollinshead *et al.*, 1999). Some IMV are transported on microtubules from virus factories to near the microtubule organizing centre where they are wrapped by two cellular membranes containing several virus proteins. This produces a triple-enveloped virus called intracellular enveloped virus (IEV). IEV are transported on microtubules to the cell surface where the outer membrane fuses with the plasma membrane to externalise a double-enveloped virion by exocytosis. This virion is called cell-associated enveloped virus (CEV) or extracellular enveloped virus (EEV) if it is released from the cell. Some authors refer to IMV, IEV and CEV/EEV as mature virus (MV), wrapped virus (WV) and extracellular virus (EV), respectively (Moss, 2006).

VACV entry is complicated by the existence of structurally distinct virions surrounded by different numbers of membranes and which bind to different receptors (Vanderplasschen & Smith, 1997). IMV enter cells by fusion with the plasma membrane (Armstrong *et al.*, 1973; Chang & Metz, 1976; Carter *et al.*, 2005) or endocytosis followed by fusion with an intracellular vesicle (Dales, 1963; Payne & Norrby, 1978; Townsley *et al.*, 2006; Townsley & Moss, 2007; Mercer & Helenius, 2008). However, for EEV a fusion event would only release a single enveloped virus into the cytosol and so replication could not start until the second lipid envelope is removed. Two solutions to this topological difficulty have been suggested. First, after endocytosis, the EEV outer envelope is disrupted by low pH within acidified vesicles, allowing the IMV to fuse with the vesicle membrane (Ichihashi, 1996; Vanderplasschen *et al.*, 1998). Second, the interaction of EEV with the cell surface induces non-fusogenic disruption of the EEV outer membrane (Law *et al.*, 2006). This rupture occurs only at the site of interaction between the plasma membrane and the virus particle, and requires cell surface glycosaminoglycans (GAGs) and the EEV proteins B5 and A34. After EEV outer membrane rupture, the internal IMV particle fuses with the plasma membrane to release a core into the cytosol. Probably, both mechanisms operate, since EEV were not ruptured on the surface of sog9 cells, which are deficient in cell surface GAGs, yet EEV still infect these cells and form plaques (Law *et al.*, 2006). Cores are then transported deeper into the cell on microtubules (Carter *et al.*, 2005).

Here the mechanism by which the EEV outer membrane is disrupted upon contact with the plasma membrane has been investigated further. We focused on the B5 protein because B5-negative EEV are resistant to GAG-mediated EEV membrane rupture (Law *et al.*, 2006).

B5 is a 42 kDa glycoprotein present in the EEV outer membrane with type I topology (Engelstad *et al.*, 1992; Isaacs *et al.*, 1992). The extracellular domain of B5 comprises four copies of a short consensus repeat (SCR) characteristic of complement control proteins (Takahashi-Nishimaki *et al.*, 1991). However, there is no evidence that B5 serves to regulate complement activity, and VACV encodes a second protein with SCRs that inhibits complement activation (Kotwal & Moss, 1988; Kotwal *et al.*, 1990). After these SCRs, B5 has an acidic stalk (ST) region before the transmembrane (TM) sequence and short cytoplasmic tail (CT). The SCRs are dispensable for targeting the B5 protein to the EEV membrane, but their deletion enhanced EEV release (Herrera *et al.*, 1998; Mathew *et al.*, 1998). The CT is dispensable for incorporation of B5 into EEV (Lorenzo *et al.*, 1998), but affects the rate of B5 transport to the cell surface (Mathew *et al.*, 2001) and recycling from the cell surface via endosomes (Ward & Moss, 2000). B5 is required for IMV wrapping to form IEV (Engelstad & Smith, 1993; Wolffe *et al.*, 1993). In addition, B5 interacts with A34 (Rottger *et al.*, 1999) and A34 is required for the efficient incorporation of B5 into IEV/EEV (Earley *et al.*, 2008; Perdiguero *et al.*, 2008).

A34 is a type II membrane protein with different glycoforms of between 23 and 28 kDa (Duncan & Smith, 1992). It is expressed late during infection and is present on the cell and EEV surface. There is an N-terminal single transmembrane domain that serves as signal sequence and membrane anchor, and an extracellular C-type lectin-like domain (Duncan & Smith, 1992). Deletion of the *A34R* gene (McIntosh & Smith, 1996) or a lys151glu mutation (Blasco *et al.*, 1993) enhanced EEV production. However, A34-negative virions have a fivefold reduced specific infectivity (McIntosh & Smith, 1996), form a small plaque (Duncan & Smith, 1992), do not form actin tails (Wolffe *et al.*, 1997; Sanderson *et al.*, 1998) and are avirulent (McIntosh & Smith, 1996).

Here we investigated the role of B5 domains in EEV membrane rupture and show that acidic residues in the ST (amino acids 241–279) proximal to the EEV membrane are critical for membrane rupture. Based on these data, a refined model for EEV entry is presented.

## METHODS

### Cells.

BSC-1, CV-1 and D98OR cells were grown in Dulbecco's modified Eagle's medium (Gibco) supplemented with 10 % fetal bovine serum (FBS) and 50 IU penicillin ml^−1^–50 μg streptomycin ml^−1^ (PS; Gibco). RK13 cells were grown in minimal essential medium (MEM, Gibco) with 10 % FBS, 2 mM l-glutamine (Gibco) and PS. BHK-21 cells were grown in Glasgow-MEM (Sigma) with 10 % FBS, 5 % tryptose phosphate broth (Sigma), 2 mM l-glutamine and PS. Alternatively, BHK-21 were grown in MEM with 10 % FBS, 81 mg MEM non-essential amino acids l^−1^ (Sigma), 2 mM l-glutamine, 1× vitamins (Gibco) and PS.

### Antibodies.

For immunoprecipitation, a rat monoclonal antibody (mAb), anti-B5 19C2 (Schmelz *et al.*, 1994), was used. To detect B5 by immunoblotting, 19C2 or a mouse mAb 36-6 were used. mAb directed against A34 (34-1), A36 [6.3, (van Eijl *et al.*, 2000)] and D8 [AB1.1, (Parkinson & Smith, 1994)] were also used. For plaque-reduction assays, Abs that neutralize IMV were either mAb 2D5 against the L1 protein (Ichihashi *et al.*, 1994) or a polyclonal mix of rabbit Abs against L1 and A27 (IMV-N serum).

### GAGs and polyions.

The following polyionic compounds were tested for their ability to rupture the EEV outer membrane: poly-dl-alanine (P9003), heparin, poly-l-aspartic acid sodium salt (P5387), poly-l-histidine (P9386), poly-l-lysine hydrobromide (P0899) and chitosan (C3646) (all from Sigma-Aldrich); dextran sulphate (31404) and poly-l-glutamic acid (81326) from Fluka. Stock solutions (10 mg ml^−1^) of these compounds were prepared in water or 1 M acetic acid (chitosan).

### Plasmids.

Mutations were introduced into the *B5R* gene by site-directed mutagenesis, PCR, splice overlap extension (Horton *et al.*, 1990) and gene cloning with the oligonucleotide primers described in Supplementary Table S1 (available in JGV Online) and DNA from VACV strain Western Reserve (WR) as template.

#### pB5R-STΔ1–19 aa.

To delete the nucleotides within the *B5R* ORF that encode the N-terminal half of the ST (amino acids 241–259 of the B5 protein, or amino acids 1–19 of the ST), oligonucleotides B5-1 and B5-3 were used to amplify the 5′ flanking region of the *B5R* ORF and the 5′ end of the *B5R* ORF up to the start of the ST. Oligonucleotides B5-4 and B5-2 were used to amplify the C-terminal half of the ST, TM, CT and the 3′ flanking region of the *B5R* ORF. Both fragments were assembled into a 1537 bp gene using splice overlap extension with oligonucleotides B5-1 and B5-2, cloned into pGEM and subcloned into *Xba*I- and *Hin*dIII-digested pSJH7 (Hughes *et al.*, 1991) to form pB5R-STΔ1–19 aa.

#### pB5R-STΔ20–39 aa.

To delete the nucleotides within the *B5R* ORF that encode the C-terminal half of the ST (amino acids 20–39), PCR was used as follows: oligonucleotides B5-1 and B5-5 were used to amplify the *B5R* 5′ flanking region and the 5′ end of the *B5R* ORF up to the N-terminal half of the ST. Oligonucleotides B5-6 and B5-2 were used to amplify the *B5R* TM, CT and the 3′ flanking region. Both fragments were assembled into a single 1533 bp gene using splice overlap extension with oligonucleotides B5-1 and B5-2, cloned into pGEM and subcloned into *Xba*I- and *Hin*dIII-digested pSJH7 (Hughes *et al.*, 1991) to form pB5R-STΔ20–39 aa.

#### Mutation of acidic residues within the B5 ST.

Virus mutants with acidic amino acids within the B5 ST replaced by alanine residues were constructed using the QuikChange PCR-based site-directed mutagenesis kit (Stratagene). A plasmid derived from pSJH7 (pB5R) containing a 2016 bp fragment encoding the entire *B5R* ORF with 429 bp upstream and 636 bp downstream was used as a template. The nine N-terminal acidic residues (amino acids 2, 3, 5, 8, 9, 12, 13, 14 and 16, Fig. 3a[Fig f3]) were converted to alanines using pB5R and primers B5-A and B5-B to form pB5R-ST2–16ala. Similarly, the five C-terminal acidic residues (amino acids 23, 28, 30, 32 and 35) were mutated to alanines using oligonucleotides B5-C and B5-D to form pB5R-ST23–35ala. Then all acidic residues within the B5 ST were changed to alanines by sequential rounds of mutagenesis using the above oligonucleotides to form pB5R-ST2–35ala. Next, one or more acidic residues adjacent to the TM domain were mutated: (i) E35A (pB5R-ST35ala) using primers B5-E and B5-F; (ii) E32A and E35A (pB5R-ST32–35ala) using primers B5-G and B5-H; (iii) E30A, E32A and E35A (pB5R-ST30–35ala) using primers B5-I and B5-J; and (iv) E28A, E30A, E32A and E35A (pB5R-ST28–35ala) using primers B5-K and B5-L; and (v) E28A and E30A (pB5R-ST28–30ala) using primers B5-M and B5-N (Fig. 4a[Fig f4]).

### Recombinant viruses.

Viruses lacking B5 SCR2-4, SCR 3-4, SCR 4 or all four SCRs and those with the B5 domains swapped with those from A56 have been described previously (Herrera *et al.*, 1998; Mathew *et al.*, 1998, 2001). Recombinant viruses containing the additional B5 ST mutations described above were made by transient dominant selection as described previously (Rodger & Smith, 2002).

### Plaque reduction assay.

Fresh EEV from the supernatant of infected cells was clarified by centrifugation for 10 min at 1000 ***g***, 4 °C. This was mixed with an equal volume of medium with or without GAGs and incubated with the IMV-neutralizing antibody (IMV-NAb) for 1 h at 37 °C. The mixture was adsorbed onto BS-C-1 cells for 90 min at 37 °C. Unbound virus and the Ab mixture were washed away and cells were overlaid with DMEM/2.5 % FBS and 1.5 % carboxymethylcellulose. The cells were incubated for 2–4 days, stained with crystal violet and the plaques were counted.

### Preparation of cell lysates and EEV lysates.

RK13 or BHK-21 cells were infected with viruses at 3–5 p.f.u. per cell for 24 h. Cells were washed in PBS, harvested and lysed with Triton/NP-40 lysis buffer (Rottger *et al.*, 1999) for 20–30 min at 4 °C. Insoluble material was centrifuged at 16 000 ***g*** for 20 min. The supernatant containing the cell extracts was frozen at −80 °C. To prepare EEV lysates, the supernatant from infected cells was harvested 24 h post-infection, centrifuged for 10 min at 1000 ***g***, 4 °C to remove detached cells and debris and then centrifuged for 2 h at 18 000 ***g***, 4 °C to collect virions. The pellet containing EEV was resuspended in Triton/NP-40 lysis buffer (Rottger *et al.*, 1999). Lysates were aliquoted and stored at −80 °C. The protein concentration of the lysates was determined by Bradford assay, using BSA as a standard.

### Immunoprecipitation.

EEV lysates were pre-cleared by incubation for at least 10 min at 4 °C with Fast Flow 4 protein G Sepharose (GE Healthcare). Lysates were then incubated with GAGs or polycations for 10 min at room temperature followed by incubation with protein G Sepharose beads cross-linked to rat mAb 19C2 (anti-B5) overnight at 4 °C. Beads were washed extensively with lysis buffer and bound proteins were eluted in 1× SDS sample buffer (50 mM Tris/HCl pH 6.8, 2 % SDS, 10 % glycerol, 0.1 % bromophenol blue, 10 mM *β*-mercaptoethanol).

### Immunoblotting.

Samples were mixed with SDS sample buffer and resolved by SDS-PAGE (15 % gel), followed by semi-dry transfer on Hybond enhanced chemiluminescence (ECL) or Hybond-P membranes (GE Healthcare). Membranes were blocked for 2–4 h at room temperature or overnight at 4 °C in PBS/0.1 % Tween20 (PBS-T) with 5 % skim milk and then incubated for 2–15 h in primary antibody diluted in PBS-T/1 % milk. After extensive washes with PBS-T, the membranes were incubated with anti-mouse-horseradish peroxidase (Sigma) 1 : 2000 in PBS-T/1 % milk for 1 h at room temperature. Membranes were washed again with PBS-T and stained with ECL+ (GE Healthcare) or home-made ECL before exposure of X-ray films.

## RESULTS

### The absence of A34 leads to a defect of B5 incorporation into EEV

**Fig. 1. f1:**
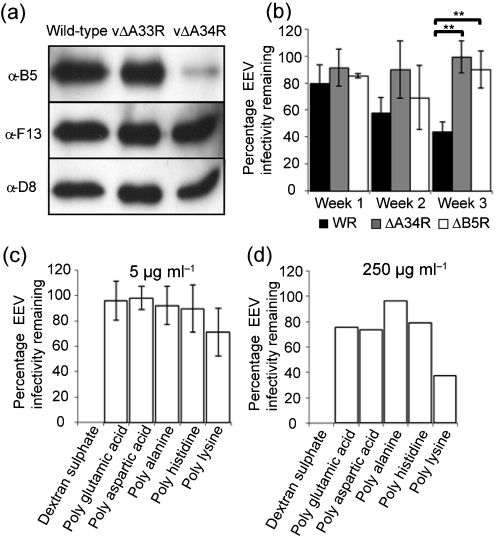
Interaction between A34 and B5 and rupture of the EEV membrane. (a) Recruitment of B5 to EEV requires the A34 protein. EEV from wild-type , vΔA33R and vΔA34R viruses were purified by sucrose density gradient centrifugation, and analysed by immunoblotting with Abs against B5, F13 and D8. (b) EEV that lacked either A34 or B5 is more stable than wild-type strain (WR). Fresh EEV were incubated with or without IMV-NAb 2D5 (1 : 2000) for 1 h at 37 °C and the infectivity was measured by plaque assay. The remaining EEV was stored at 4 °C and retitrated on days 7, 14 and 21. The black bars and asterisks indicate a significant difference (*P*<0.01) between the groups, as determined by Student's *t*-test. (c and d) Ionic polypeptides did not affect EEV membrane integrity. Fresh EEV was incubated with or without poly-l-aspartic acid (*M*_r_ 5000–15 000), poly-l-glutamic acid (*M*_r_ 2000–15 000), poly-d-lysine (*M*_r_ 70 000–150 000), poly-l-histidine (*M*_r_ 5000–250 000) poly-dl-alanine (*M*_r_ 1000–5000) and dextran sulphate (positive control) at 5 μg ml^−1^ (c) or 250 μg ml^−1^ (d) in presence of mAb 2D5 (diluted 1 : 1000). Samples were incubated for 1 h at 37 °C. The total infectivity prior to treatment, the infectivity that is resistant to IMV-NAb, and the infectivity that is resistant to IMV-NAb in the presence of PA were determined by plaque assay. Results are expressed as a percentage of infectivity compared with the sample incubated with anti-IMV NAb. Data shown are the mean±sd of two experiments (c) or one representative experiment out of three (d). In all experiments, EEV was also incubated with polyions in the absence of IMV-Nab to show that the compounds did not affect infectivity directly (results not shown).

EEV lacking either B5 or A34 are resistant to GAG-induced disruption (Law *et al.*, 2006). This indicated that either both proteins were needed, or that loss of one protein affected incorporation of the other. Accordingly, we purified EEV from wild-type (WT) virus, or viruses lacking gene *A34R* or *A33R* and analysed the protein composition by immunoblotting. The IMV surface protein D8 and the EEV protein F13 were present at equal levels in all viruses, but B5 was reduced considerably in the absence of A34, but not A33 (Fig. 1a[Fig f1]). These data are in accord with other observations that A34 is required for the efficient recruitment of B5 to the EEV membrane (Earley *et al.*, 2008; Perdiguero *et al.*, 2008).

### The EEV membrane is more stable in the absence of A34 or B5

To test if loss of either B5 or A34 affected EEV membrane stability, fresh EEV was produced with WR, vΔB5R (Engelstad & Smith, 1993) and vΔA34R (McIntosh & Smith, 1996). A fraction of each virus was incubated with and without IMV-NAb followed by plaque assay to determine the proportion of virus with an intact EEV membrane. The remaining supernatant was stored at 4 °C for 7, 14 and 21 days and then was retitrated as before. Fig. 1(b) shows that the proportion of WT (WR) EEV with an intact outer membrane decreased with time whilst the proportion of vΔB5R and vΔA34R remained relatively constant. Thus, in the absence of B5 or A34 (which also results in the reduction of B5) the EEV membrane is more stable than that of the WT.

### GAG-mediated rupture of the EEV membrane is affected by their charge and structure

Incubation of EEV with polyanions (PAs) such as heparin (HP) or dextran sulphate (DS) disrupts the EEV membrane and makes virions sensitive to IMV-NAb (Law *et al.*, 2006). To ascertain the relative importance of the ionic charge and carbohydrate structure of PAs in this process, WT VACV strain WR EEV was incubated with a range of ionic compounds comprised of amino acid subunits. The anionic compounds poly-l-glutamic acid and poly-l-aspartic acid (described previously by Law *et al.*, 2006) were compared with cationic compounds poly-l-histidine and poly-d-lysine, and with poly-dl-alanine and DS as controls. As expected, treatment of EEV with 5 μg DS ml^−1^ and IMV-NAb reduced EEV infectivity to below the limit of detection (4×10^5^ p.f.u. ml^−1^); however, none of the other poly-ionic compounds had a substantial effect on EEV infectivity (Fig. 1c[Fig f1] and Law *et al.*, 2006). The molecular masses of these compounds varied considerably, producing different charge to mass ratios, and this might affect EEV membrane stability. To address this, EEV was incubated with a much higher concentration (250 μg ml^−1^) of these compounds. At this concentration, DS and IMV-NAb inhibited all EEV infectivity whilst the other compounds had little effect (Fig. 1d[Fig f1]). The effect observed in the presence of poly-d-lysine is unclear because it also reduces IMV infectivity (data not shown). Collectively, these data suggest that both the negative charge and the carbohydrate structure of GAGs are required for efficient EEV membrane rupture.

### B5 TM and ST are involved in GAG-induced rupture of the EEV membrane

**Fig. 2. f2:**
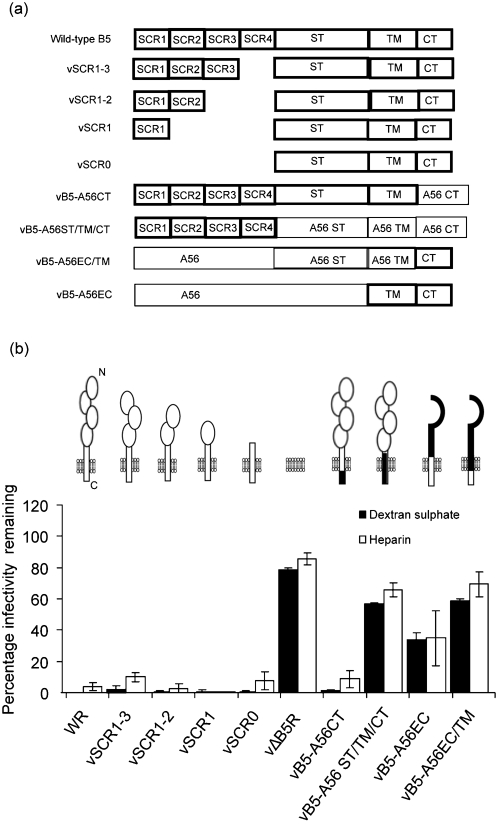
Structure of B5 mutant proteins and the inhibition of EEV infectivity by dextran sulphate (DS) and heparin (HP). (a) Illustration of B5 mutants. B5 regions are highlighted by thick lines. (b) Fresh EEV was incubated with medium or medium containing DS or HP (5 μg ml^−1^) in presence of mAb 2D5. Samples were processed and data re-expressed as described in Fig. 1. Data shown are the mean±sd of three experiments.

To investigate which B5 domain(s) is (are) involved in EEV membrane rupture, EEV produced by a panel of viruses with mutated B5 proteins (Fig. 2a[Fig f2]) were tested for their sensitivity to GAG-mediated membrane rupture. Some of the viruses have one or more SCRs deleted (Herrera *et al.*, 1998; Mathew *et al.*, 1998), others have sections of B5 substituted with the equivalent regions of the VACV WR A56 (haemagglutinin, HA) protein (Mathew *et al.*, 2001). Viruses with one or more SCR deleted or with the CT swapped with the A56 CT had the same phenotype as WT WR and were sensitive to PA-induced EEV membrane disruption (Fig. 2b[Fig f2]), suggesting that the ST or TM regulates EEV membrane rupture. Consistent with this, the TM is implicated because vBR-A56ST/TM/CT is resistant to GAGs. This is supported further by vB5-A56EC (in which the BR SCRs are replaced by the ectodomain, EC, of A56) and vB5-A56EC/TM (in which all B5 except the CT is replaced by A56). The presence of B5 CT alone (vB5-A56EC/TM) does not make the membrane sensitive to GAGs, whereas the presence of B5 TM (and CT) (vB5-A56EC) has an intermediate phenotype. Collectively, these B5 mutants indicate that the B5 SCRs and CT are not involved in EEV membrane rupture.

### Role of the B5 ST acidic residues in the EEV entry process

**Fig. 3. f3:**
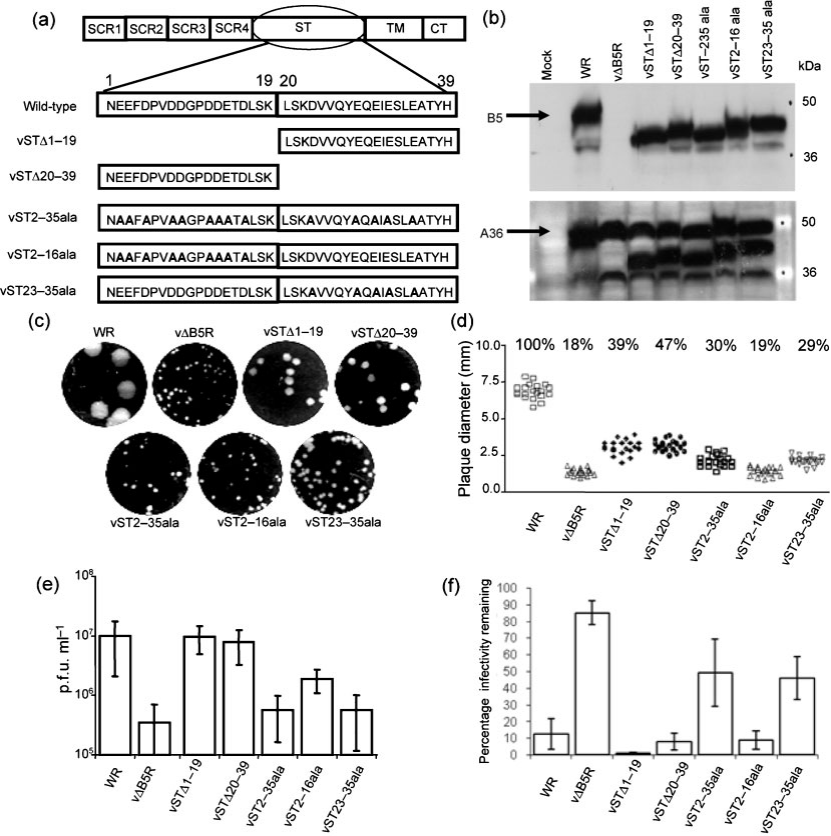
Mutational analysis of the B5 ST. (a) Mutations incorporated into the B5 ST (amino acids 1–39). (b) Immunoblot of the B5 and A36 proteins from infected cell lysates. BHK-21 cells were infected at 3 p.f.u. per cell and incubated for 24 h at 37 °C. The cells were lysed and the proteins were separated by SDS-PAGE (12 % gel). (Top) The membrane was probed with anti-B5 mAb 19C2. WT B5 (arrow) is 42 kDa. (Bottom) The membrane was then reprobed with anti-A36 mAb. A36 is approximately 50 kDa (arrow). (c) Plaque phenotype. BS-C-1 cell monolayers were infected and overlaid with DMEM/2.5 % FBS/1.5 % CMC. After a 10 day incubation period at 37 °C, cell monolayers were stained with crystal violet and scanned using a GelDoc imager. (d) Plaque size comparison. The diameter of 20 plaques of each virus from two independent experiments in (c) was measured and the percentage plaque size compared with that of WT WR plaques was calculated. (e) EEV titres: BHK-21 cells were infected at 3 p.f.u. per cell for 24 h and the supernatant was harvested, incubated with mAb 2D5 (diluted 1 : 2000) for 1 h at 37 °C and the infectivity was measured by plaque assay. Data shown are the mean±sd of at least three experiments. (f) Effect of HP on EEV membrane disruption, as in Fig. 2(b).

To investigate the role of B5 ST in EEV membrane dissolution, virus mutants with a modified ST were generated. The ST is highly acidic with 14 of the 39 residues being aspartic or glutamic acid. These acidic residues were treated as two clusters, membrane proximal (ST amino acids 1–19) and membrane distal (ST amino acids 20–39), and mutations were introduced as shown in Fig. 3(a). Before determining the effect of mutations on EEV membrane stability, it was important to characterize their effect on B5 expression and EEV morphogenesis. B5 expression was confirmed by immunoblotting (Fig. 3b[Fig f3]). WT B5 is 42 kDa, whilst the modified B5 proteins resolved at between 38 and 41 kDa in SDS-PAGE due to deletion, substitution or change in pI. The control protein A36 was expressed by all viruses, including vΔB5R.

To characterize the growth of these mutant viruses, their ability to form plaques (Figs 3c and d[Fig f3]) and produce EEV (Fig. 3e[Fig f3]) was studied. vΔB5R makes tiny plaques compared with WT. The plaques formed by vSTΔ1–19 and vSTΔ20–39, lacking either the membrane distal or proximal half of the stalk region, respectively, were smaller than those made by WR, but noticeably larger than those of vΔB5R. In contrast, plaques formed by vST2–35ala, vST2–16ala and vST23–35ala were smaller. vST2–16ala produced plaques of similar size to those of vΔB5R, whereas those of v2–35ala and vST23–35ala were marginally larger. Collectively, these data showed that the ST and the associated acidic residues affect virus plaque size.

Measurement of EEV production by the different mutants (Fig. 3e[Fig f3]) showed that vΔB5R produces 5–10-fold less EEV than WT, as noted previously (Engelstad & Smith, 1993). Deletion of either half of the B5 ST had little effect on EEV production, and the EEV titres of vST2–35ala, vST2–16ala and vST23-35ala were all intermediate between the wild-type and vΔB5R levels. Notably >70 % of virus infectivity in the culture medium of cells infected by each new mutant was resistant to IMV-NAb and so represented EEV. Actin tail and CEV formation were studied by immunofluorescence as described previously (Herrero-Martinez *et al.*, 2005) and confirmed that vSTΔ1–19 and vSTΔ20–39 were similar to WT, whilst vST2–35ala, vST2–16ala and vST23–35ala produced actin tails and CEV in reduced amounts (data not shown). These data show that deletion of either half of the B5 ST had little effect on virus morphogenesis, whereas a greater effect was found with alanine substitution of the acidic residues. The EEV sensitivity of the mutants to GAGs was investigated by plaque reduction assay (Fig. 3f[Fig f3]). HP disrupted the EEV membranes of vSTΔ1–19, vSTΔ20–39 and vST2–16ala so that these mutants were sensitive to IMV-Nab, whilst EEV of vST2–35ala and vST23–35ala was resistant to HP. The results suggest that acidic residues proximal to the EEV membrane are crucial because the first three mutants retain membrane-proximal acidic residues, whereas these residues are eliminated from the last two mutants.

**Fig. 4. f4:**
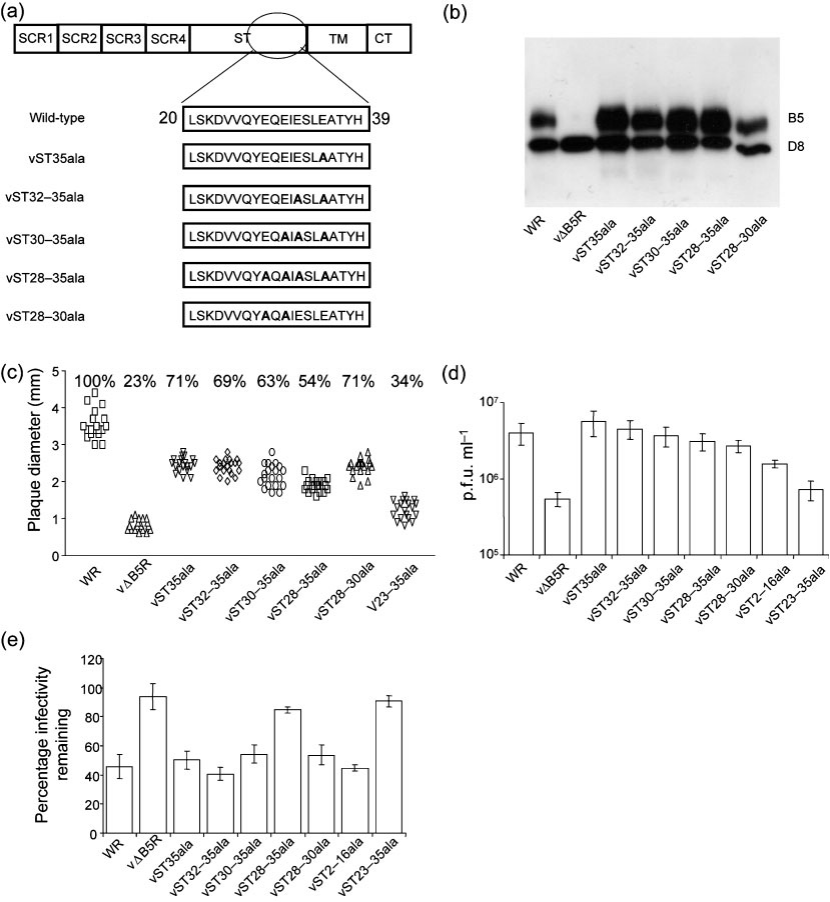
Mutations in the C-terminal region of the B5 ST affected plaque size, EEV titre and sensitivity to HP. (a) Alanine substitutions in the B5 ST. (b) Western blot of the B5 and D8 proteins. Infected cell lysates were prepared, processed and analysed by immunoblotting as in Fig. 3b[Fig f3] except that anti-D8 mAb was used. (c) Plaque size analysis as in Fig. 3. (d) EEV production as in Fig. 3e[Fig f3]. The IMV NAb used was the IMV-N-serum (diluted 1 : 100) rather than mAb 2D5. (e) Effect of HP on EEV membrane disruption. See Fig. 2(b) for details except that IMV-N serum (1 : 100) was used instead of 2D5.

To determine how near to the TM the acidic residues need to be for EEV membrane disruption, a second panel of alanine substitution viruses was created in which the acidic residues near to the TM were changed sequentially to alanine (Fig. 4a[Fig f4]). As before, the expression of these mutant proteins and the effects of these mutations on plaque size and EEV formation were characterized. B5 expression was confirmed by immunoblotting (Fig. 4b[Fig f4]), with expression of the VACV D8 protein as a control. The plaques of these mutants were 54–71 % the size of the WT plaques, but bigger than vΔB5R and vST23–35ala described above (Fig. 4c[Fig f4]), and they produced similar amounts of EEV to WT (Fig. 4d[Fig f4]). These mutants also produced actin tails and CEV at levels similar to WT (data not shown). Incubation of EEV with HP disrupted the EEV membranes of all these viruses, except for vST28–35ala, which, like vΔB5 and vST23–35ala, was resistant (Fig. 4e[Fig f4]). For this assay IMV-N-serum was used and this did not neutralize IMV as efficiently as mAb 2D5. Data shown in Figs 3 and 4 indicate that an acidic residue within 11 aa of the predicted TM region of EEV membrane determines the sensitivity of EEV membrane to GAGs.

### Effect of polyanions and polycations on B5–A34 interaction

**Fig. 5. f5:**
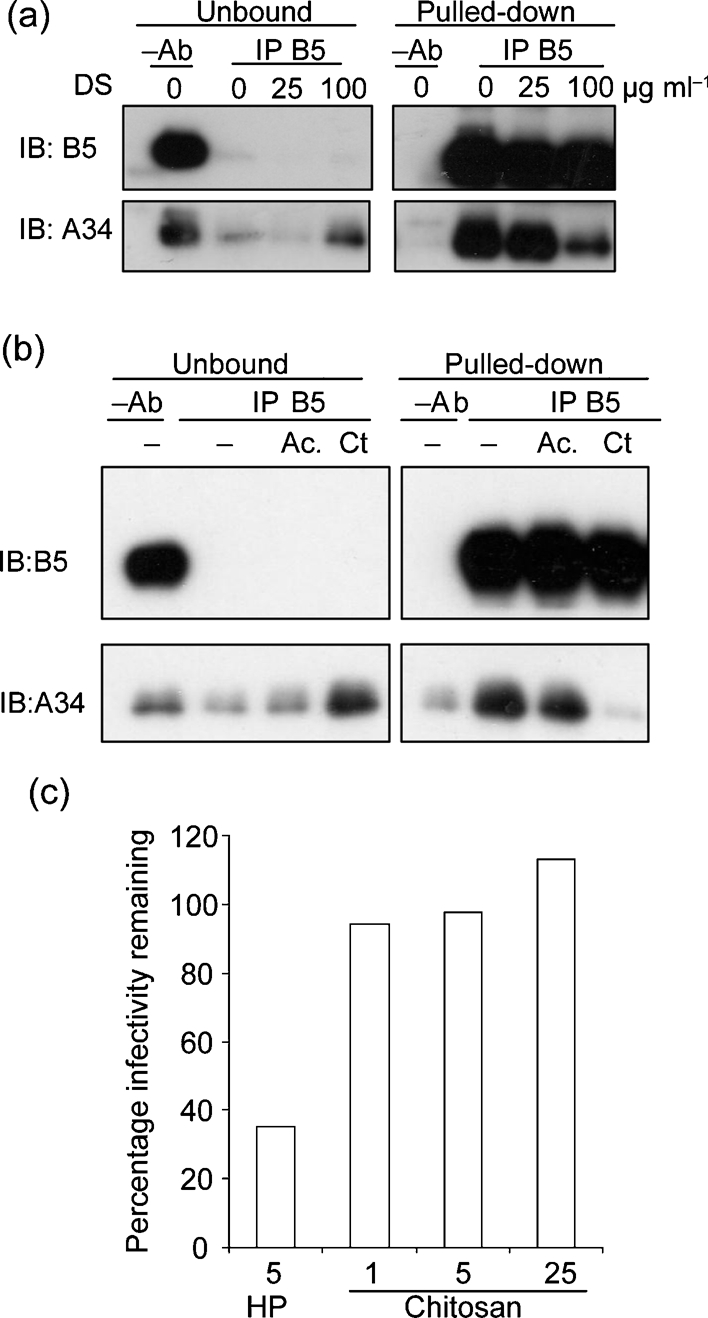
Ionic compounds interfere with the A34–B5 interaction. (a) GAGs. IHD-J EEV extracts were incubated with protein G beads with or without cross-linked anti-B5 mAb 19C2 in the presence or absence of DS. Supernatants (1/40 of the total volume) and proteins bound to the beads (1/10) were analysed by immunoblotting using mouse mAbs against B5 or A34. (b) Polycations. EEV extracts were incubated with protein G beads or protein G beads cross-linked to mAb 19C2 in presence or absence of chitosan (Ct) at 25 μg ml^−1^. Chitosan had to be solubilized in 1 M acetic acid, and so a control incubated with acid alone (Ac.) was included. Immunoprecipitation, electrophoresis and immunoblotting were performed as in (a). (c) Fresh EEV was incubated with medium or medium containing chitosan (Ct) at 1, 5 or 25 μg ml^−1^ or HP at 5 μg ml^−1^ as a control in the presence of IMV-N serum (1 : 100). Samples were incubated for 1 h at 37 °C, and virus infectivity was determined by plaque assay. Data shown are from one representative experiment out of four.

B5 is acidic with an isoelectric point (pI) of 4.7 whilst A34 is basic (pI 9.6) so the interaction of these proteins (Rottger *et al.*, 1999) may be electrostatic. If so, their interaction might be disrupted by charged molecules and this might contribute to destabilization of the EEV membrane. To test if GAGs and other charged compounds affected the interaction between A34 and B5, we performed co-immunoprecipitation experiments using B5–A34 complexes prepared from EEV (Fig. 5a[Fig f5]). VACV strain IHD-J was used because this strain produces higher levels of EEV (Payne, 1979). B5 was immmunoprecipitated from EEV lysates with anti-B5 mAb and, as expected, a proportion of A34 co-precipitated with B5. Incubation with increasing concentrations of DS (Fig. 5a[Fig f5]) or HP (data not shown) reduced the amount of A34 pulled-down with B5 and increased A34 in the unbound fraction. This shows that GAGs disrupt the B5–A34 interaction. Moreover, negative charge is needed because desulphated HP did not break A34–B5 interaction (data not shown).

If A34 and B5 interact electrostatically, polycations might also disrupt the B5–A34 complex. Accordingly, we tested the effect of chitosan, a positively charged carbohydrate derived from chitin and with structural similarity to GAGs. This showed that the interaction of A34 and B5 was greatly reduced by this polycation (Fig. 5b[Fig f5]). However, unlike PAs (Fig. 1), this polycation was unable to rupture the EEV membrane, because the virions remained resistant to IMV-NAb (Fig. 5c[Fig f5]).

## DISCUSSION

Previously, it has been shown that EEV can enter cells following rupture of the outer membrane induced by contact with GAGs on the cell surface and that this phenomenon requires proteins A34 and B5 (Law *et al.*, 2006). Here we show that the requirement for the A34 protein is indirect and that in its absence considerably less B5 protein is incorporated into EEV. Similar observations have been made by others (Earley *et al.*, 2008; Perdiguero *et al.*, 2008), and an earlier report also showed reduced B5 incorporation in the absence of A34, although this was not commented on (McIntosh & Smith, 1996). Data presented also demonstrate that the outer membrane of EEV lacking either B5 or A34 is more stable than that of the WT and, for vΔA34, this is consistent with its resistance to disruption by polyanions (Law *et al.*, 2006) and interaction with cell surfaces (Husain *et al.*, 2007). A34-negative EEV has lower specific infectivity, but freeze–thawing resulted in an increase in infectivity due to release of IMV (McIntosh & Smith, 1996). In view of these observations our study of EEV outer membrane disruption has focussed on B5.

By using a panel of VACV mutants lacking different B5 domains, we showed that the extracellular SCRs and the CT are dispensable for GAG-mediated membrane rupture. This implied that the TM and/or ST are required. It remains unclear whether the TM is involved directly, but the ST certainly is. This region is highly negatively charged at neutral pH (14 out of 39 residues are aspartic or glutamic acid) and data presented show that negative charges in this region are critical for GAG-mediated membrane rupture. More specifically, acidic residues proximal to the viral membrane are important. If either the membrane proximal or membrane distal half of the ST is deleted the membrane remains sensitive to GAG-mediated rupture because in both cases acidic residues remain close to the virion membrane. However, if the acidic residues within the membrane-proximal region were changed to alanines, the membrane became resistant to GAG-mediated rupture. In contrast, if the membrane distal acidic residues were mutated to alanines the membrane remained sensitive. All viruses with a mutated ST produced plaques smaller than WR and larger, or the same size, as vΔB5R, but plaque size did not correlate with sensitivity to GAG-mediated membrane rupture. This is evident from Fig. 3, where vΔB5R and vST2–16ala have equivalent sized plaques, but the EEV differ in the sensitivity to GAG-mediated membrane rupture. Conversely, vSTΔ20–39 and vST2–16ala have differently sized plaques, but the same sensitivity to membrane rupture. Further mutagenesis defined how close to the membrane acidic residues need to be to enable GAG-mediated membrane rupture. Rupture still occurred if an acidic residue was ≤11 residues from the TM, but not if the distance was 16 residues. Also, the vST28–30ala mutant shows that two acidic residues close to the membrane are sufficient. Overall, these results demonstrate that the negative charge of the B5 ST is critical for membrane rupture.

Given the basic nature of A34 (pI 9.6), electrostatic interactions with the B5 acidic stalk are possible. Recently, Perdiguero *et al.* (2008) showed that interactions with A34 can be mediated by the extracellular domain of B5 and also the TM/CT/ST. Our data are consistent with the latter possibility. In addition, Perdiguero *et al.* (2008) and Earley *et al.* (2008) showed that the extracellular domain of A34, which contains most of the basic residues, is responsible for binding to B5. Further mutagenesis and structural determination of the complex is required to define precisely how these proteins interact.

An electrostatic interaction between B5 and A34 might be disrupted by large positive or negative compounds and we showed that A34 co-precipitation with B5 was decreased by PAs (e.g. GAGs) and polycations like chitosan (Fig. 5) or poly-lysine (not shown). However, unlike PAs, the polycation could not induce membrane rupture and we propose this is because after it disrupts the A34–B5 complex it remains bound to B5 and neutralizes its negative charge. In contrast, the disruption of the A34–B5 complex by PAs would leave the negatively charged residues of B5 exposed close to the membrane. These membrane-proximal acidic residues are critical for membrane disruption. A model that might explain this requirement is electrostatic repulsion between the negatively charged B5 stalks and the negatively charged phospholipid head groups, leading to the destabilization and rupture of the membrane.

In conclusion, we demonstrate that, without A34, less B5 is incorporated into the EEV membrane and EEV lacking either B5 or A34 have more stable outer membranes. The acidic B5 ST is critical for the rupture of the EEV membrane and the acidic residues must be within 11 aa of the virion membrane to induce membrane instability.

## Supplementary Material

[Supplementary Table]
