# Supplementary material for: Acidic residues in the membrane-proximal stalk region of vaccinia virus protein B5 are required for glycosaminoglycan-mediated disruption of the extracellular enveloped virus outer membrane
Source: J Gen Virol. 2009 Jul;90(Pt 7):1582–91. doi: 10.1099/vir.0.009092-0 (PMC2885056; doi:10.1099/vir.0.009092-0)
Supplement: [Supplementary Table] [file supp_90_7_1582__index.html]

 Acidic residues in the membrane-proximal stalk region of vaccinia virus protein B5 are required for glycosaminoglycan-mediated disruption of the extracellular enveloped virus outer membrane -- Roberts et al. 90 (7): 1582 Data Supplement - Supplementary Table -- Journal of General Virology

### Acidic residues in the membrane-proximal stalk region of vaccinia virus protein B5 are required for glycosaminoglycan-mediated disruption of the extracellular enveloped virus outer membrane, by Kim L. Roberts, Adrien Breiman, Gemma Carter, Helen Ewles, Michael Hollinshead, Mansun Law and Geoffrey L. Smith

*Journal of General Virology* vol. **90**, part 7, pp. 1582–1591

**Supplementary Table S1.** Primers used for construction of B5 mutants [PDF] (101 KB)

  
  
