# Supplementary material for: Acidic residues in the membrane-proximal stalk region of vaccinia virus protein B5 are required for glycosaminoglycan-mediated disruption of the extracellular enveloped virus outer membrane
Source: J Gen Virol. 2009 Jul;90(Pt 7):1582–91. doi: 10.1099/vir.0.009092-0 (PMC2885056; doi:10.1099/vir.0.009092-0)
Supplement: [Supplementary Table] [file supp_90_7_1582__1.pdf]

Supplementary Table S1. Primers used for construction of B5 mutants

Restriction enzyme sites are underlined. Amino acid substitutions are shown in bold type.

**Name**	**Sequence (5′–3′)**
B5-1	TGCTCTAGAGCACTAGTCTCGACGTATACG
B5-2	CCCAAGCTTGGGCAACGCTTACAGAAACATCGC
B5-3	GTACAACGTCTTTCGAGAGAGTTCGTACACATATTGG
B5-4	CCAATATGTGTACGAACTCTCTCGAAAGACGTTGTAC
B5-5	GTTAACGCCACTATGATTATTTTGCTCAAATCTGTCTC
B5-6	GAGACAGATTTGAGCAAAATAATCATAGTGGCGTTAAC
B5-A	GAACTAACG**C**AG**C**ATTTG**C**TCCAGTGG**C**TG**C**TGGTCCCG**C**CG**C**TG**C**GACAG**C**TTTGAGCAAACTC
B5-B	CAAA**G**CTGTC**G**CA**G**CG**G**CGGGACCA**G**CA**G**CCACTGGA**G**CAAAT**G**CT**G**CGTTAGTTCGTACACATAT
B5-C	CTCGAAAG**C**CGTTGTACAATATG**C**ACAAG**C**AATAG**C**ATCGTTAG**C**AGCAACTTATCATATAATC
B5-D	AGTTGCT**G**CTAACGAT**G**CTATT**G**CTTGT**G**CATATTGTACAACG**G**CTTTCGAGAGTTTGCTC
B5-E	GTACAATATGAACAAGAAATAGAATCGTTAG**C**AGCAACTTATC
B5-F	GATAAGTTGCT**G**CTAACGATTCTATTTCTTGTTCATATTGTAC
B5-G	GTACAATATGAACAAGAAATAG**C**ATCGTTAG**C**AGCAACTTATC
B5-H	GATAAGTTGCT**G**CTAACGAT**G**CTATTTCTTGTTCATATTGTAC
B5-I	GTACAATATGAACAAG**C**AATAG**C**ATCGTTAG**C**AGCAACTTATC
B5-J	GATAAGTTGCT**G**CTAACGAT**G**CTATT**G**CTTGTTCATATTGTAC
B5-K	GTACAATATG**C**ACAAG**C**AATAG**C**ATCGTTAG**C**AGCAACTTATC
B5-L	GATAAGTTGCT**G**CTAACGAT**G**CTATT**G**CTTGT**G**CATATTGTAC
B5-M	GTACAATATG**C**ACAAG**C**AATAGAATCGTTAGAAGCAACTTATC
B5-N	GATAAGTTGCTTCTAACGATTCTATT**G**CTTGT**G**CATATTGTAC

Roberts, K. L., Breiman, A., Carter, G., Ewles, H., Hollinshead, M., Law, M. & Smith, G. L. (2009). Acidic residues in the membrane-proximal stalk region of vaccinia virus protein B5 are required for glycosaminoglycan-mediated disruption of the extracellular enveloped virus outer membrane. *J Gen Virol* **90**, 1582–1591.
